# The factors of quality of life among patients after myocardial infarction in Poland: a cross-sectional study. The quality of life among patients after myocardial infarction

**DOI:** 10.1038/s41598-024-65525-z

**Published:** 2024-07-10

**Authors:** Ewelina Kolarczyk, Dominika Kohanová, Agnieszka Witkowska, Marek Szymiczek, Agnieszka Młynarska

**Affiliations:** 1 Department of Gerontology and Geriatric Nursing, Faculty of Health Sciences, Medical University of Silesia, Katowice, Poland; 2 Department of Nursing, Faculty of Social Sciences and Health Care, Constantine the Philosopher University, Nitra, Slovak Republic; 3 Department of Cardiology, Electrotherapy and Angiology, Scanmed S.A. Racibórz Medical Center, 47-400 Racibórz, Poland

**Keywords:** Quality of life, Myocardial infarction, Chronic illness, Chronic disease, Myocardial ischemia, Cardiology, Health care, Medical research

## Abstract

The quality of life (QoL) is now recognised as a central indicator of the effectiveness of interventions especially in patients after myocardial infarction (MI). The QoL may be important predict poor outcomes in cardiac patients.The present work aims to increase knowledge of the level of QoL in patients after MI. Moreover, the paper analyses the QoL in relation to sociodemographic factors and the degree of functioning in chronic disease. The study was conducted among 231 patients who were hospitalized due to MI within the period of June 2021 to June 2022 in the Hospital in Racibórz in Poland. The WHO Quality of Life Questionnaire and the Chronic Disease Functioning Scale were used. The analysis showed a statistically significant correlation (coefficient value 0.5 <|r/rho|≤ 0.7) between general functioning in chronic disease and the average QoL (rho = 0.56; p < 0.001)and somatic QoL levels(rho = 0.52; p < 0.001), as well as a moderately strong positive correlation with the QoL level on the psychological domain (rho = 0.50; p < 0.001), social domain(rho = 0.48; p < 0.001) and environmental domain (rho = 0.43; p < 0.001). The results of this study suggested that healthcare workers adopts appropriate policies for the implementation of quality of life, which can reduce the number of repetitive referrals to the hospital and costs imposed on the health system.

## Introduction

The concept of quality of life has been defined by the World Health Organization (WHO) as "an individual's perception of their position in life in the context of the culture and value systems in which they live and in relation to their goals, expectations, standards and interests"^1^. In recent years, attention has been given to the role of quality of life in the impact of disease and treatment on functioning in the physical, mental and social spheres. In clinical practice, the assessment of individual parameters of quality of life improves the effectiveness of therapy and is particularly important in the care of patients with chronic diseases^2,3^. Cardiovascular diseases, due to their prevalence as well as social and economic effects, are a special group in which the assessment of quality of life should be performed. However, since quality of life, according to the WHO definition, covers all aspects of human life, medical sciences more often use the concept of health-related quality of life (HRQoL). HRQoL represents a multidimensional concept that examines the physical, emotional and social impact of diseases on patients' lives^2^. In medicine, apart from objective clinical parameters, the impact of therapy and disease on a patient's quality of life is increasingly assessed, which allows for the consideration of a patient's point of view in the assessment^4^.

Despite advances in pharmacological and interventional treatment, ischaemic heart disease(IHD) is still the leading cause of death due to cardiovascular diseases^5–7^. There is preliminary evidence that the detrimental impact of work stress on health is independent of conventional risk factors for IHD and their treatment^6^. Myocardial infarction (MI), which is the most common manifestation of IHD, is one of the leading causes of death from chronic diseases worldwide^8,9^. A previous scientific study confirmed that there is a relationship between poorer quality of life and a 3.6-fold higher risk of death as a consequence of IHD^10^. It has been shown that the type of cardiac surgery is associated with the deterioration of functional status and HRQoL among elderly patients hospitalized for acute myocardial infarction (AMI).Patients who underwent coronary artery bypass grafting (CABG) and percutaneous coronary intervention (PCI) had lower HRQoL levels than those treated pharmacologically^11^. In contrast, data from studies by Tegn et al. showed no clinically significant differences in HRQoL after 1 year between older MI patients randomized to undergo an invasive strategy compared to a conservative approach^12^.

Predictive factors that reduce the quality of life of MI patients include obesity and smoking^13^. It is believed that the comprehensive cardiac rehabilitation of patients after MI makes it possible to significantly improve all aspects of quality of life, especially the sphere of physical health among younger people and the sphere of mental health among older people^13^. However, despite numerous studies on HRQoL, knowledge about all its factors among patients after MI remains limited^14^. According to the current standards, the goal of stable coronary artery disease therapy is to eliminate cardiovascular risk factors and improve long-term prognosis and quality of life. Deterioration of functional status, assessed using HRQoL or the scale of functioning in activities of daily living (ADL), is associated with an increase in mortality in this group of patients^15,16^. Therefore, evaluating a patient's quality of life after MI may provide health care professionals with the opportunity to individualize treatment advice for secondary prevention. Identification of HRQoL factors can help to identify patients who are at risk of low HRQoL at the stage of convalescence or rehabilitation after myocardial infarction^17^.

This study aimed to examine the level of quality of life in patients after myocardial infarction in relation to sociodemographic factors and the degree of functioning in chronic disease. The main research aims were to determine the following: (1) the level of quality of life inpatients after MI; (2) whether and which sociodemographic factors affect the quality of life of MI patients (in the somatic, psychological, social and environmental spheres); and (3) whether and how the quality of life of patients after MI affects functioning in chronic disease (in terms of the overall outcome, impact of the disease on the patient, impact of the patient on the disease, and impact of the disease on the patient's attitudes).

## Methods

### Study design

A cross-sectional, single-centre design was utilized in this study. The study was carried out according to the STROBE checklist for observational studies^18^.

### Settings and participants

The study used a purposefully selected group of patients (n = 231) from the Unit of Cardiology, Electrotherapy and Angiology in the Silesian Voivodeship in Racibórz Hospital in Poland. The patients were hospitalized due to MI within the period of June 2021 to June 2022. Written consent for the management of the Scanmed S.A. was obtained for the study. The minimum sample size was 196 patients, which was calculated based on the available patient population with a 95% confidence interval. The data to determine the minimum number of individuals within the groups were obtained from the demographic situation in Poland up to 2020^19^. The questionnaires were provided in a paper form (“pen-pencil questionnaire”), and the interview was conducted by two investigators who were medical staff on the ward and one investigator who was a researcher from a university. The inclusion criteria for the study were as follows: (1) adult participants diagnosed with MI, as well as STEMI andNSTEMI; (2) patients treated with PCI; (3) patients who provided informed consent to participate in the study; (4) patients without dementia-related disorders; and (5)patients without mental disorders. Patients who did not consent to participate in the study or who were unable to answer questions due to hearing disorders, vision disorders, advanced senile dementia or diagnosed mental illnesses were excluded from the study. Data collection began in January 2020 but was suspended during the COVID-19 pandemic because the ward was reclassified as a COVID-19 ward. Surveys that were collected on the ward before the pandemic were disposed of as part of the disinfection of the ward. Twenty-three people refused to participate in the survey, and 36 surveys were not included in the statistical analysis because the survey subscales contained missing responses.

### Instruments

The research was carried out using two standardized measures: the WHO Quality of Life Questionnaire(WHOQOL BREF) and the Chronic Disease Functioning Scale (FCIS). Moreover, an original survey on sociodemographic and clinical factors was used.

The WHO Quality of Life Questionnaire (WHOQOL BREF) is a person-centred, multilingual instrument for subjective assessment and is designed for generic use as a multidimensional profile, enabling the comparison of patients with a wide range of diseases and conditions. It is an integrated26-item version of the WHOQOL-100 that contains items in four domains physical health, psychological, social relations, and environment, with all facet items scored as part of their hypothesized domain. Cronbach’s internal consistency values are acceptable (> 0.7) for Domains 1, 2 and 4 of the scale, i.e., physical health (0.82), psychological (0.81), environment (0.80), and social relationships (0.68)^20^.The questionnaire was adapted to Polish conditions and was shown to be a reliable tool for assessing quality of life^21^.

The Chronic Disease Functioning Scale (FCIS) allows results to be obtained for four scales: general functioning of the patient in the illness, the impact of the illness on the patient, the impact of the patient on the illness and the impact of the illness on the patient's attitudes. The internal consistency of the questionnaire expressed by a Cronbach coefficient was 0.855, indicating its high reliability and homogeneity. The set of items divided into 3 subscales allows for the evaluation of the impact of the disease on the patient, the patient’s impact on the disease and the impact of the disease on the patient’s attitudes^22^.

### Statistical analyses

The statistical analysis was performed at e-statystyka.com.pl, a company specializing in statistical calculations in the fields of medicine, psychology, pedagogy, sociology and other scientific fields. The analysis used a p < 0.05 to indicate significance. Parametric tests (Student's T test or ANOVA) or their nonparametric equivalents (Mann‒Whitney U test or Kruskal‒Wallis test) were used to analyse quantitative variables, which were broken down into groups. Correlations amongthe variables were verified using Pearson’s (r) or Spearman’s (rho) correlation coefficient. The selection of tests was made on the basis of the distribution of the variables, which was verified by the Shapiro‒Wilk test. Calculations were made in the statistical software R ver.3.6.0, PSPP program and MS Office 2019.

### Ethical procedure

The study was conducted under the recommendations of the Helsinki Declaration reported by the World Medical Association^23^ and the guidelines of Good Clinical Practice^24^. Before beginning the study, the respondents were informed about the anonymous and voluntary nature of the survey. Consent to participate in the study was obtained from each respondent. The study protocol was approved by the Bioethics Committee at the Medical University of Silesia in Katowice on04 March 2019 (ethical approval code: KNW/0022/KB/46/19).Informed consent was obtained from all subjects involved in the study.

## Results

### Characteristics of the study group

The sample consisted of 231 patients (76 women and 155 men), and detailed information on the characteristics of the study group is provided in Table 1.

**Table 1:** Characteristics of the study group.

Variable		*N*	*%*
Gender	Female	76	32.9%
	Male	155	67.1%
Age	≤ 40 years	9	3.9%
	41–50 years	21	9.2%
	51–60 years	50	21.8%
	61–70 years	81	35.4%
	≥ 70 years	68	29.7%
BMI	Underweight	2	0.9%
	Normal weight	55	23.8%
	Overweight	94	40.7%
	First-degree obesity	60	26.0%
	Second-degree obesity	12	5.2%
	Extreme obesity	8	3.5%
Marital status	Widowed	40	17.9%
	Married	161	71.9%
	Divorced	14	6.3%
	Single	9	4.0%
Dwelling place	City	124	60.1%
	Village	82	39.9%
Education level	Primary	39	17.1%
	Vocational	93	40.9%
	Secondary	74	32.5%
	Higher	21	9.2%
Professional activity	Working	68	38.2%
	Unemployed	12	6.7%
	Pensioner	98	55.1%
Children	Yes	202	92.6%
	No	16	7.4%
Comorbidities	Hypertension	152	65.8%
	Diabetes	69	29.8%
	Lipid disorders	56	24.2%
	Other diseases	70	30.3%
	No	7	3.03%
Smoking	Currently	61	26.4%
	Never	112	48.4%
	In the past	58	25.1%
Myocardial infarction	First episode	192	83.4%
	Subsequent episode	38	16.6%

### Evolution of the quality of life of MI patients

The median values of the quality of life results obtained using the WHOQOL questionnaire, presented in sten values (1–10), ranged from 5.44 to 5.53, which means that the level of quality of life of the entire study group, for each of the spheres, was average. The table below shows the distribution of quality of life levels in the study group. Detailed information can be found in Tables 2 and 3.

**Table 2:** Descriptive statistics of quality of life in patients after myocardial infarction.

Quality of life	*N*	*M* ± *SD*	*Me*	*Q1–Q3*
Average quality of life—points	231	68.66 ± 14.84	70.00	59.00–80.00
Domain 1: Physical health—points	231	61.45 ± 17.79	63.00	50.00–75.00
Domain 2: Psychological—points	231	71.33 ± 16.50	69.00	59.50–81.00
Domain 3: Social relations—points	231	71.52 ± 19.66	75.00	56.00–81.00
Domain 4: Environment—points	231	70.98 ± 15.74	75.00	63.00–81.00
Average quality of life—sten	231	5.52 ± 2.02	6.00	4.00–7.00
Domain 1: Physical health—sten	231	5.51 ± 1.91	6.00	4.00–7.00
Domain 2: Psychological—sten	231	5.53 ± 2.08	5.00	4.00–7.00
Domain 3: Social relations—sten	231	5.44 ± 1.95	6.00	4.00–6.00
Domain 4: Environment—sten	231	5.5 ± 2.03	6.00	4.00–7.00

**Table 3:** Distribution of average quality of life levels and quality of life in individual spheres.

Quality of life		*N*	*%*
Average quality of life	Low	65	28.1%
	Intermediate	88	38.1%
	High	78	33.8%
Domain 1: Physical health	Low	71	30.7%
	Intermediate	100	43.3%
	High	60	26.0%
Domain 2: Psychological	Low	75	32.5%
	Intermediate	63	27.3%
	High	93	40.3%
Domain 3: Social relations	Low	60	26.0%
	Intermediate	119	51.5%
	High	52	22.5%
Domain 4: Environment	Low	82	35.5%
	Intermediate	68	29.4%
	High	81	35.1%

### The sociodemographic and clinical variables of quality of life in MI patients

**Figure 1:**
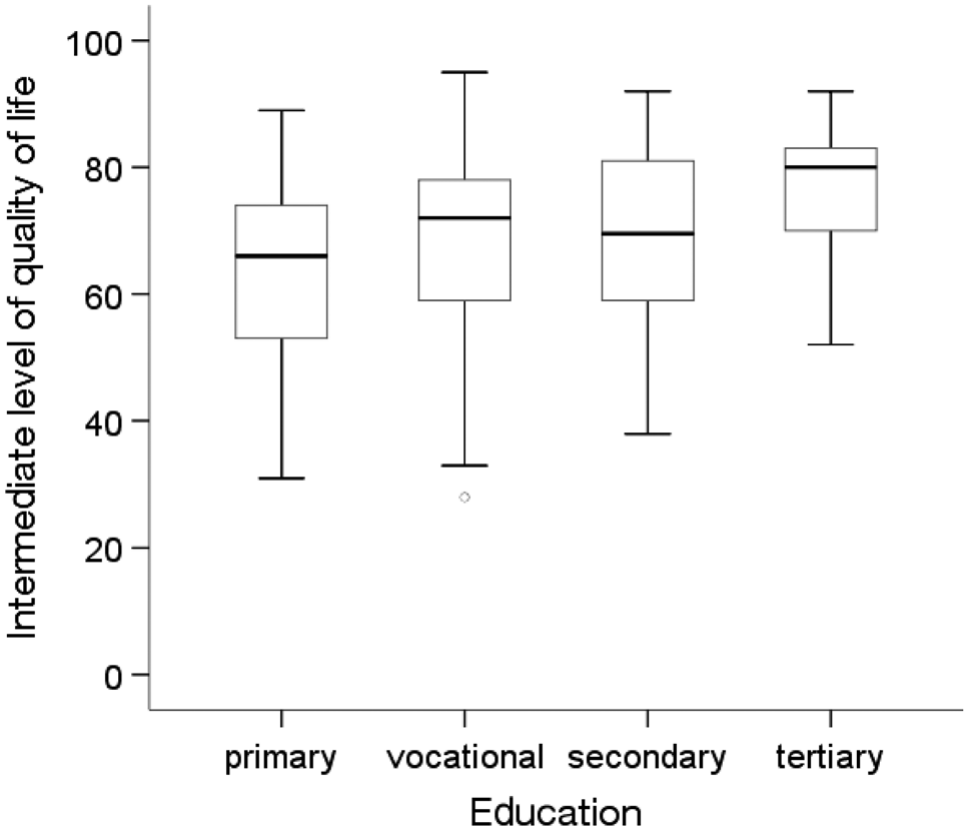
Effects of education level on quality of life in MI patients.

**Figure 2:**
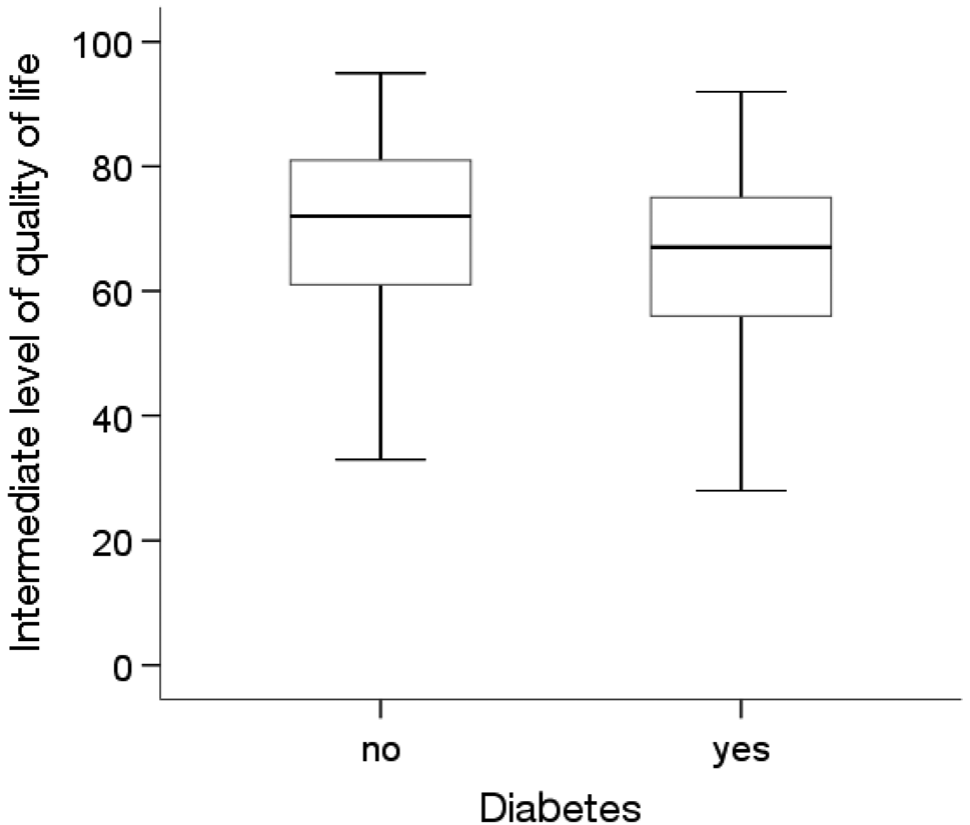
Effects of diabetes on quality of life in MI patients.

**Figure 3:**
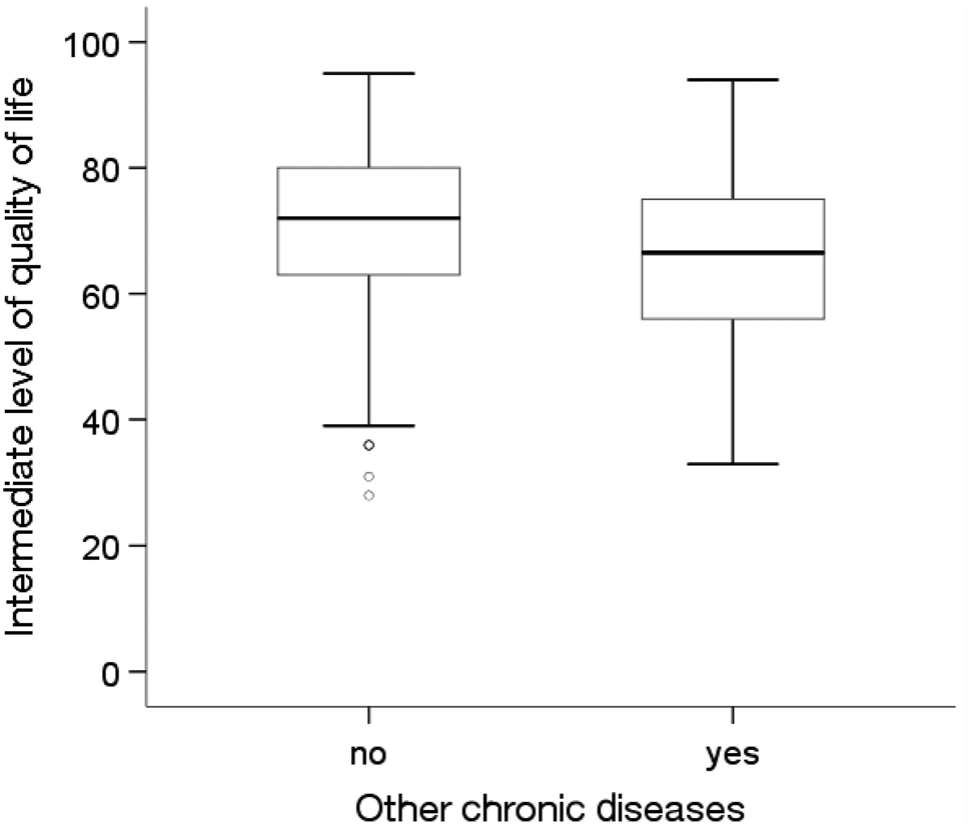
Effects of other chronic diseases on quality of life in MI patients.

The analysis showed statistically significant differences in the average quality of life level depending on education level and the prevalence of diabetes and other chronic diseases(p < 0.05). The Kruskal‒Wallis test showed that people with higher education levels (Me = 80.00) had a statistically significantly higher (p = 0.012) average quality of life than people with a primary education (Me = 66.00) (Fig. 1). The Mann‒Whitney U test showed that people without diabetes (Me = 72.00) and without other chronic diseases (Me = 72.00) were characterized by a significantly higher average quality of life than people with diabetes (Me = 67.00; p = 0.006) and other chronic diseases (Me = 66.50; p = 0.027) (Figs. 2 and 3).

**Figure 4:**
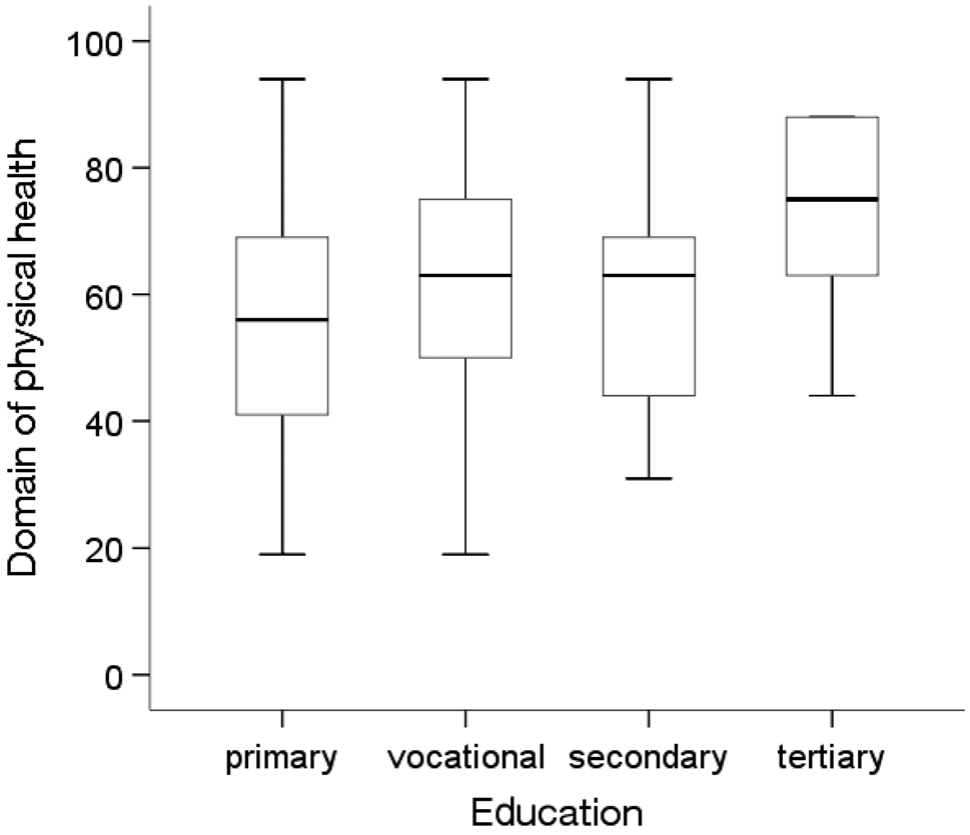
Effects of education level on quality of life in the physical domain in MI patients.

**Figure 5:**
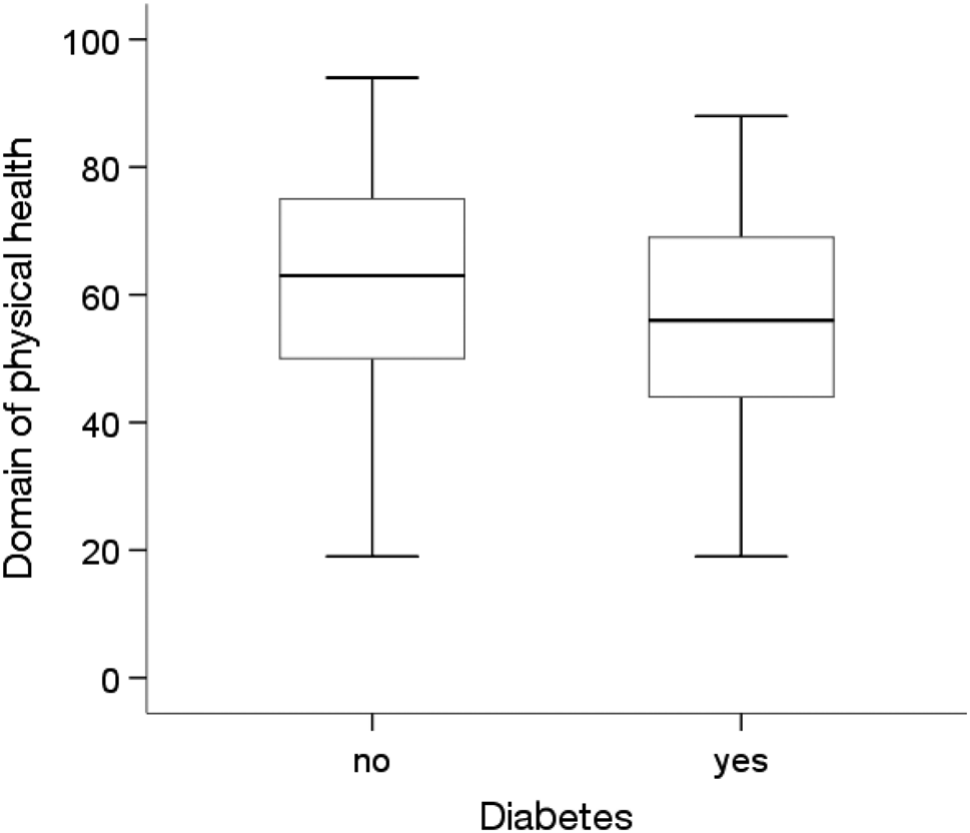
Effects of diabetes on quality of life in the physical domain in MI patients.

**Figure 6:**
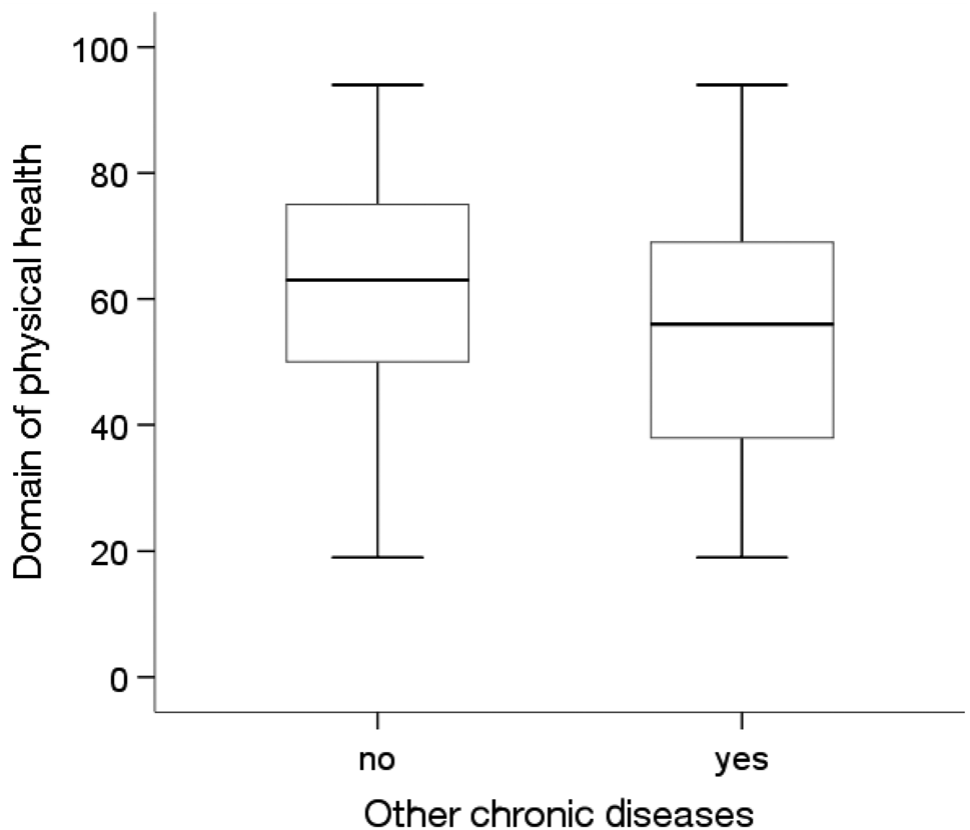
Effects of other chronic diseases on quality of life in the physical domain in MI patients.

The Mann‒Whitney U test showed statistically significant differences in quality of life in the physical health domain depending on education level and the prevalence of diabetes and other chronic diseases (p < 0.05). People with higher education levels (Me = 75.00) were characterized by a statistically significantly higher quality of life (p = 0.003) in the physical health domain than people with primary (Me = 56.00) and secondary (Me = 63.00) education (Fig. 4). In addition, people without diabetes (p = 0.005) and other chronic diseases (Me = 63.00;p = 0.001) were characterized by a significantly higher quality of life in this area than people with these diseases (Me = 56.00)(Figs. 5 and 6).

**Figure 7:**
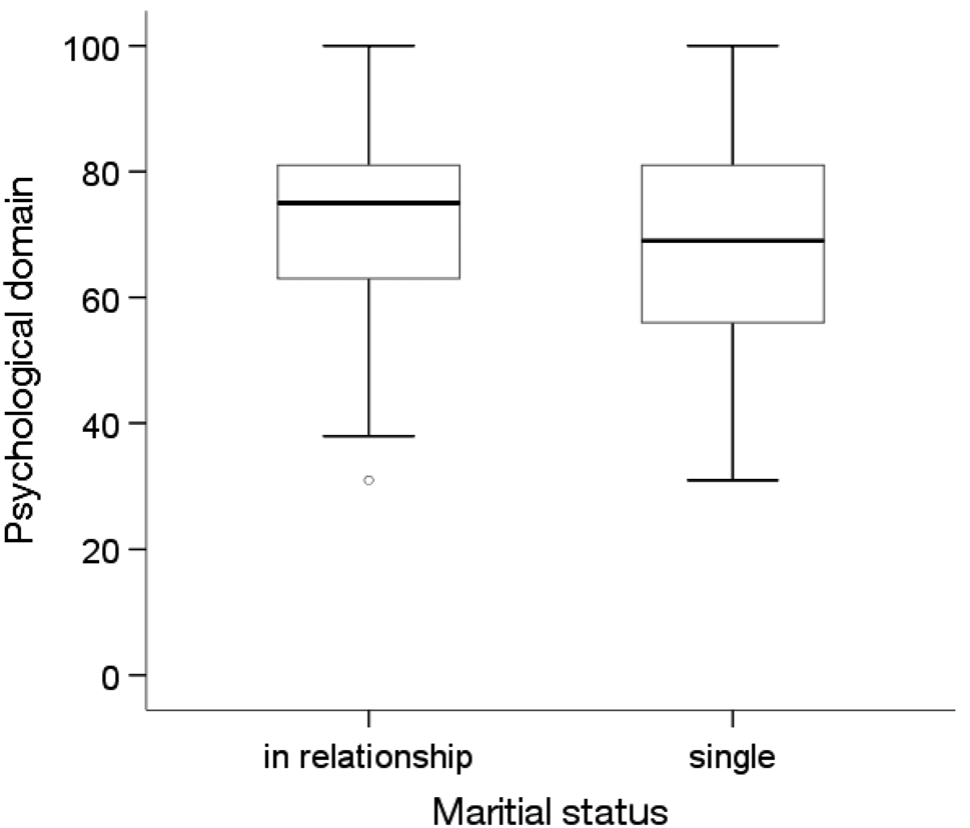
Effects of marital status on quality of life in the psychological domain in MI patients.

**Figure 8:**
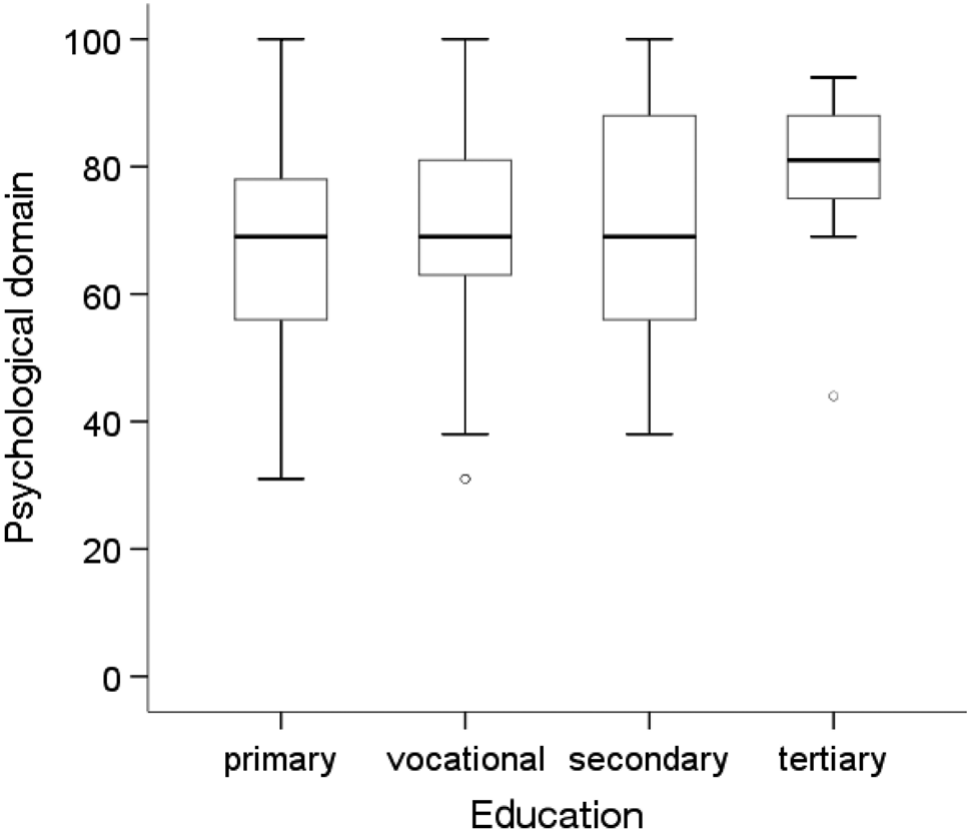
Effects of education on quality of life in the psychological domain in MI patients.

**Figure 9:**
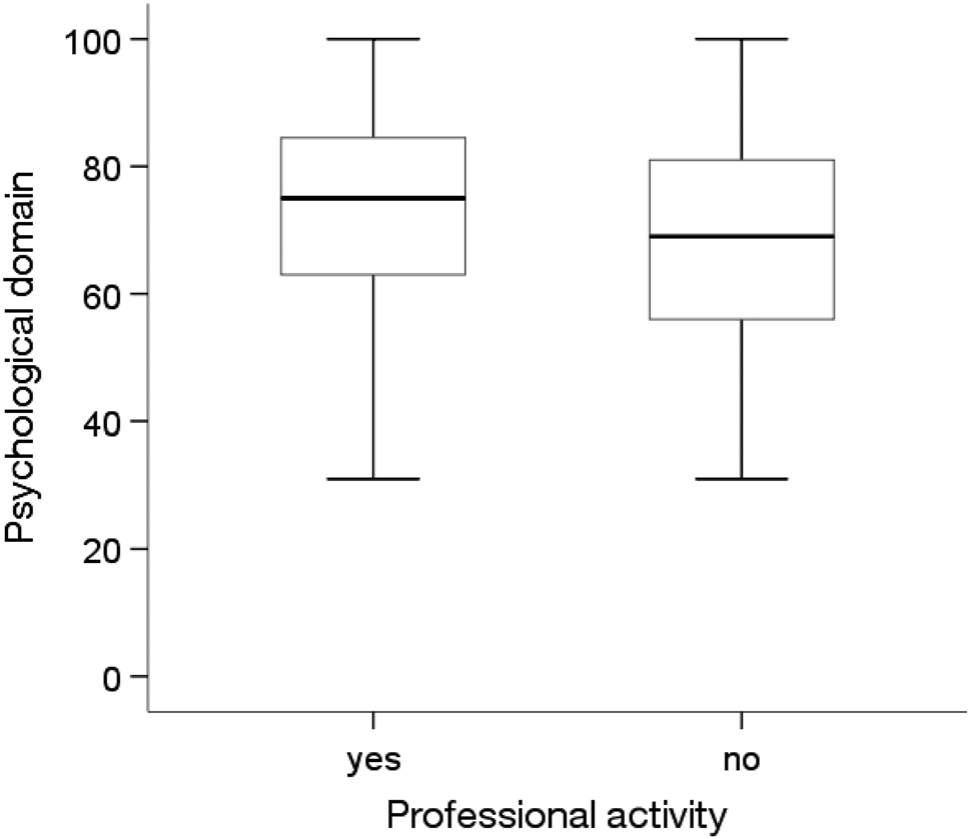
Effects of professional activity on quality of life in the psychological domain in MI patients.

**Figure 10:**
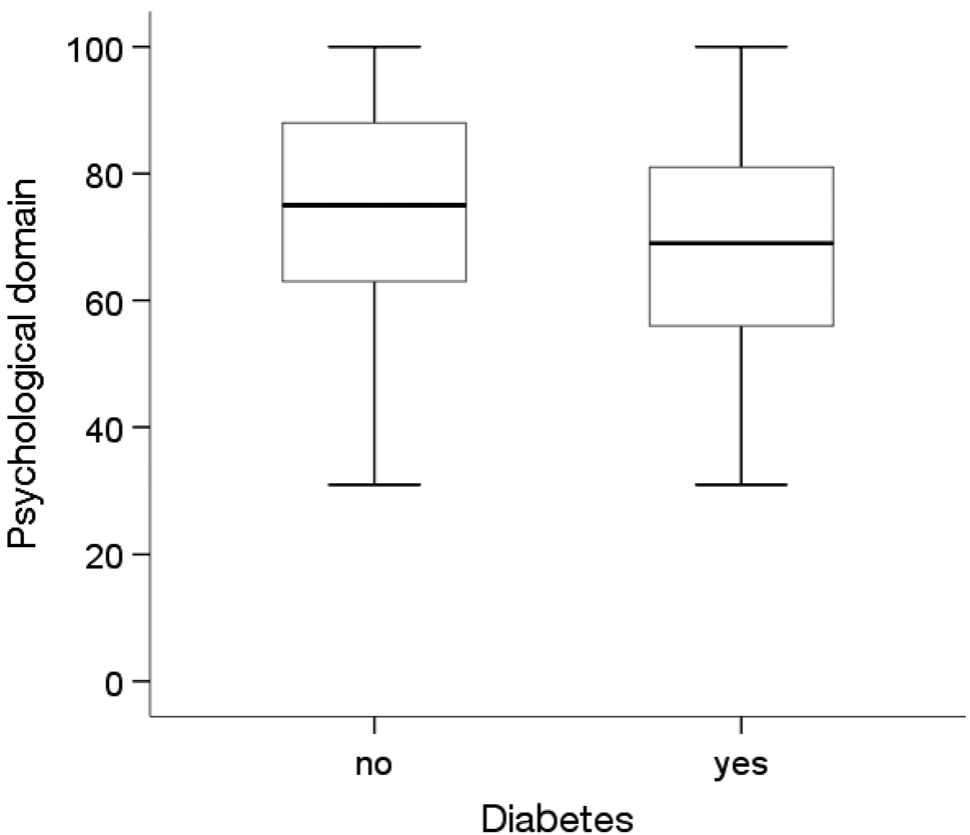
Effects of diabetes on quality of life in the psychological domain in MI patients.

According to the Mann‒Whitney U test, there were significant differences in the quality of life in the psychological domain, depending on marital status, education level, professional activity and the presence of diabetes(p < 0.05). People who were in a relationship (Me = 75.00, p = 0.046), had a higher education level (Me = 81.0; p = 0.010), were professionally active (Me = 75.00; p = 0.047) and did not have diabetes (Me = 75.00; p = 0.004) showed significantly higher quality of life in the psychological domain than people who were unmarried, had a primary education, were economically inactive and had diabetes (for these four groups, an Me = 69.00 was obtained) (Figs. 7, 8, 9 and 10).

**Figure 11:**
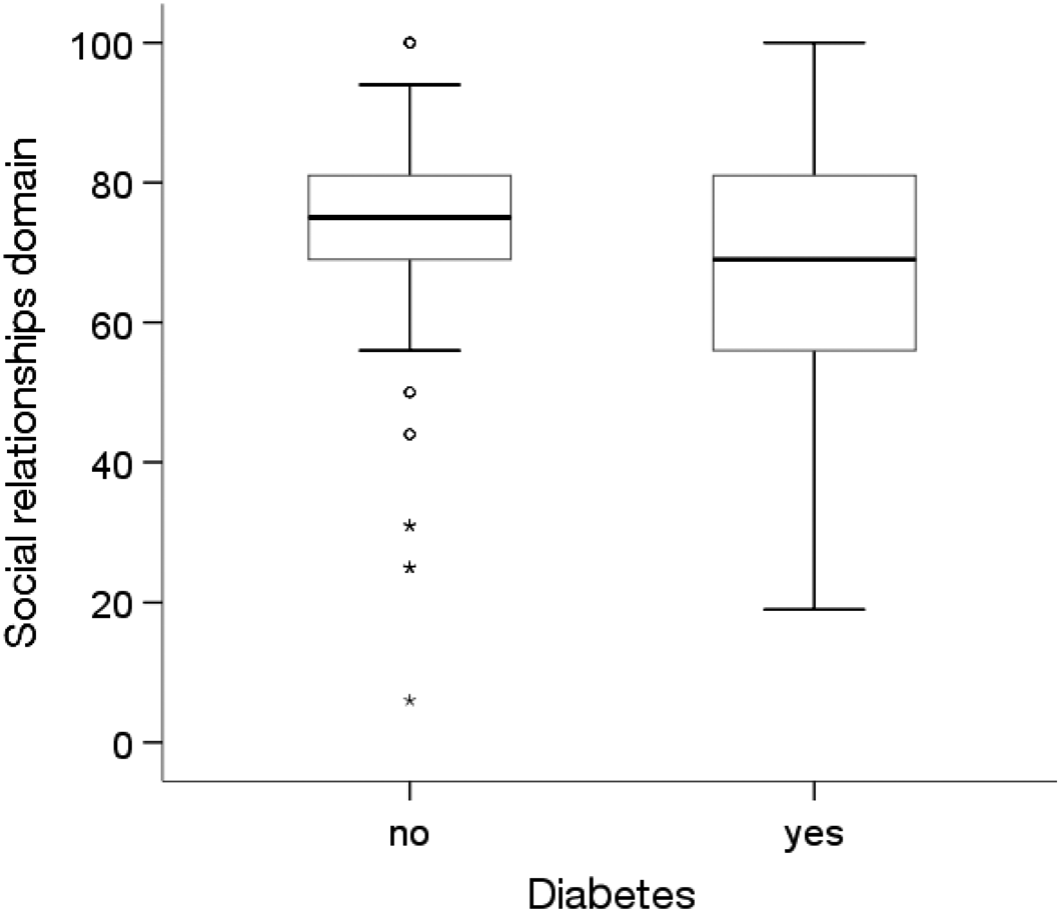
Effects of diabetes on quality of life in the social domain in MI patients.

**Figure 12:**
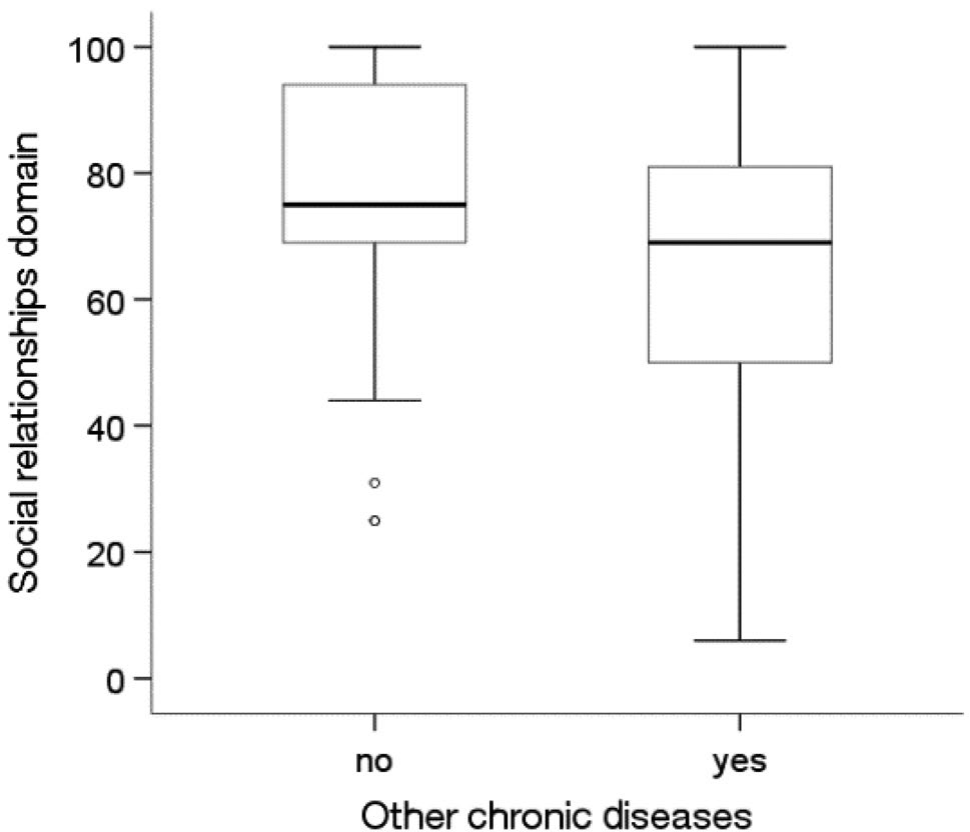
Effects of other chronic diseases on quality of life in the social domain in MI patients.

There were significant differences in the quality of life in the social domain depending on the prevalence of diabetes and other chronic diseases (p < 0.05).Among people without diabetes (p = 0.019) and other diseases (Me = 75.00; p = 0.035), the quality of life in the social domain was statistically significantly higher than that among people with these diseases (Me = 69.00) (Figs. 11 and 12).

Statistically insignificant differences in the quality of life in the environmental domain resulting from the analysed sociodemographic and clinical variables were found (p > 0.05). Quality of life was slightly higher among women, people aged over 70 years, people of normal weight, people who were in a relationship, people residing in the city, people with a higher education level, and people who were professionally active. Quality of life was also slightly higher among people with children, people without comorbidities, people who did not smoke at all, and people with a subsequent heart attack. Detailed information is included in Table 4.

**Table 4:** The quality of life in the environmental domain in relation to sociodemographic and clinical variables in patients after MI.

Variable		*N*	*M* ± *SD*	*Me*	*Q1–Q3*	*p*
Sex	Female	76	71.16 ± 16.57	75.00	56.00–81.00	0.8302
	Male	155	70.90 ± 15.37	75.00	63.00–81.00	
Age	≤ 60 years	80	69.53 ± 15,51	75.00	56.00–81.00	0.5874
	61–70 years	81	71.28 ± 16.62	75.00	63.00–81.00	
	≥ 70 years	68	72.59 ± 15.12	75.00	63.00–84.50	
BMI range	Normal	55	71.24 ± 17.69	75.00	56.00–88.00	0.8854
	Overweight	94	71.11 ± 16.03	75.00	63.00–81.00	
	Obesity	80	70.56 ± 14.27	75.00	63.00–81.00	
Marital status	In a relationship	161	71.99 ± 15.08	75.00	63.00–81,00	0.0722
	Single	63	67.38 ± 17.39	69.00	56.00–81.00	
Place of residence	City	124	70.36 ± 14.87	69.00	59.50–81.00	0.879^2^
	Village	82	69.74 ± 16.94	75.00	56.00–81.00	
Education level	Primary	39	66.64 ± 17.47	63.00	56.00–81.00	0.220^4^
	Vocational	93	71.67 ± 15.23	75.00	63.00–81.00	
	Secondary	74	71.46 ± 15.55	75.00	56.00–88.00	
	Higher	21	75.62 ± 14.01	81.00	69.00–81.00	
Professional activity	Active	68	70.53 ± 14.83	75.00	56.00–81.00	0.576^2^
	Inactive	110	68.90 ± 15.60	69.00	63.00–81.00	
Children	Yes	202	70.96 ± 15.85	75.00	63.00–81.00	0.438^2^
	No	16	66.50 ± 17.52	72.00	56.00–81.00	
Arterial hypertension	No	79	72.37 ± 14.99	75.00	63.00–81.00	0.352^2^
	Yes	152	70.26 ± 16.12	69.00	63.00–81.00	
Diabetes	No	162	72.31 ± 15.15	75.00	63.00–81.00	0.066^2^
	Yes	69	67.87 ± 16.75	69.00	56.00–81.00	
Lipid disorders	No	175	71.10 ± 15.72	75.00	63.00–81.00	0.928^2^
	Yes	56	70.63 ± 15.97	75.00	56.00–81.00	
Other chronic diseases	No	161	71.27 ± 15.95	75.00	63.00–81.00	0.707^2^
	Yes	70	70.31 ± 15.34	72.00	63.00–81.00	
Smoking	Currently	61	70.51 ± 16.38	75.00	56.00–81.00	0.911^3^
	Never	112	71.53 ± 15.79	75.00	63.00–81.00	
	In the past	58	70.43 ± 15.19	69.00	63.00–81.00	
Myocardial infarction	First	192	70.55 ± 15.91	75.00	59.50–81.00	0.370^2^
	Next	38	72.74 ± 14.93	75.00	69.00–81.00	
Total		231	70.98 ± 15.74	75.00	63.00–81.00	–

### Correlation between quality of life and functioning with chronic illness

The analysis using Spearman's correlation coefficient showed a statistically significant (p < 0.05) correlation (coefficient value 0.5 <|r/rho|≤ 0.7) between general functioning in chronic disease and average quality of life (rho = 0.56; p < 0.001) and somatic quality of life (rho = 0.52; p < 0.001), as well as a moderately strong positive correlation with quality of life in the psychological (rho = 0.50; p < 0.001), social (rho = 0.48; p < 0.001) and environmental domains (rho = 0.43; p < 0.001).This means that the better a patient’s general functioning in disease was, the higher their quality of life in all dimensions.

There was also a statistically significant, strong positive correlation between the impact of the disease on the patient and the average quality of life(rho = 0.56; p < 0.001) and physical health quality of life (rho = 0.56; p < 0.001) and a moderately strong, positive correlation with the quality of life in the psychological (rho = 0.49; p < 0.001), social(rho = 0.46; p < 0.001) and environmental domains (rho = 0.38; p < 0.001).The higher the scores of the patient’s impact on the disease was, the higher their quality of life in all its dimensions.

The impact of the disease on the patient's attitude was significantly and positively correlated with average quality of life (rho = 0.57; p < 0.001) and quality of life in the psychological domain (rho = 0.53; p < 0.001)and moderately strong and positively correlated with the somatic quality of life (rho = 0.49; p < 0.001), social (rho = 0.49; p < 0.001) and environmental domains(rho = 0.47; p < 0.001). It was therefore shown that the lower the impact of the disease on the patient's attitudes was (the higher the score), the higher their quality of life in all dimensions.

## Discussion

This study examined the quality of life of patients after MI and the relationship between sociodemographic and clinical factors and functioning in chronic illness. The study showed that the sociodemographic variables that affect quality of life are education level (overall scores and somatic and psychological domains), the presence of diabetes (overall scores and somatic, psychological and social domains) and the presence of other chronic diseases (overall scores and somatic, psychological and social domains)status, as well as marital status and professional activity in the psychological domain. The above results are in line with the study conducted by Endalew et al. in Ethiopia^25^, who found that the level of education among patients after MI affected health-related quality of life in each domain. Contrary to this study, Endalew et al. noted the impact of place of residence on the level of health-related quality of life. When comparing the studies described above, the age and cultural differences of the groups in the study by Endalew et al. and in this study should be taken into account.

However, the role of professional activity was also noticed in a study by Hawkes et al., who found that the quality of life was lower in post-MI patients who were unemployed^26^. This is consistent with the results of this study. A 6-month observation in the study by Gąsiecka et al. showed that a higher baseline BMI and the presence of dyslipidaemia increased the quality of health in patients after myocardial infarction^27^. Contrary to the study by Gąsiecka et al., in this study, BMI and hypercholesterolemia did not affect the quality of life of patients after MI^27^. In contrast, this study showed that the presence of diabetes and other chronic diseases reduced the quality of life of patients after MI.A cross-sectional study conducted in Spain by Rodríguez-Almagro et al. showed an average level of quality of life among people with diabetes, similar to the results of our study conducted among patients after MI^28^. Diabetes is a disease, and the course of diabetes is characterized by the development of chronic complications that contribute to the coexistence of other comorbidities. In the present study, the presence of other chronic diseases correlated with the quality of life of patients after MI. The obtained results can be compared to a study conducted by Kolarić et al., who analysed the quality of life of patients with diabetes in relation to the occurrence of chronic complications. The authors of these studies suggest that differences in the assessment of the quality of life of diabetes patients depend on the type of chronic complication^29^.

This study indicated an average quality of life in 88(38.1%) patients, while 78(33.8%) patients were characterized by a high level, and 35(28.1%) exhibited a low quality of life. Studies conducted by Endalew et al.^25^ in a group of Ethiopian patients after MI and studies conducted by Mollon et al.^30^ in a group of American patients showed a low level of quality of life in this group of patients. Previous research results on the subject indicate different data on the level of quality of life in relation to the age of the respondents. According to Arnold et al.^31^, older age was associated with better quality of life in post-MI patients, and studies by Oninska-Bulik^32^ found that older age predicted poor quality of life. Studies by Mori et al. showed that over half of the elderly patients (≥ 75 years) hospitalized for MI within 6 months of hospitalization had a significant decrease in at least 1 of the 3 functional domains of the ADL daily functioning scale and HRQoL^11^. According to these authors, the risk of decline was lower in patients treated with PCI or CABG compared to other medical treatments^10^. In this study, 68 people (29.4%) aged ≥ 75 years underwent PCI, and the average quality of life was measured during hospitalization. The analysis of the results of this study obtained different results from the abovementioned studies because age did not correlate with the level of quality of life inpatients after MI.

Hawkes et al. showed that a higher level of anxiety correlated with a lower level of quality of life in patients after MI^26^. Research conducted by Oginska-Bulik showed that among patients after MI, people with a type D personality were characterized by a lower quality of life compared to people with an atypical type D personality^32^. Džubur et al. showed the influence of depression on decreased quality of life among post-MI patients^33^. Similar results were obtained by Spanish researchers Upadhyayi et al., who observed that depression and poor social support led to impairments in the physical and social domains of QoL^34^. In this study, the correlation of the psychosocial variable, i.e., the impact of functioning in chronic disease, on the quality of life of patients after myocardial infarction was examined. In this paper, in the field of psychosocial factors, the impact of a patient’s functioning after MI was examined, and to our knowledge, the impact of this factor on the quality of life of patients after MI has not been investigated thus far. This study showed that the lower the impact of disease on a patient and the lower the impact of disease on a patient's attitudes is, the higher their quality of life in all its dimensions. According to French et al., who investigated the relationship between disease perceptions and quality of life in post-MI patients, therapeutic interventions aimed at changing disease perceptions may be useful in improving HRQoL after MI^35^. A change in a patient's perception of their illness, which affects their quality of life in terms of health, may translate into their satisfaction with the medical services provided. This is confirmed in a study by Steele et al., who showed that patients with an optimistic attitude better assessed the effectiveness of the therapy and more willingly followed the doctor's recommendations^36^.

The main limitation of our study is its single-centre design and the selection of patients from only one region in Poland (Silesian Region), which might limit the generalizability of the results. In addition, the limitations of this work include missing survey answers and the long period of data collection. The observational study design does not allow conclusions to be drawn regarding causality when identifying possible factors that improve or reduce HRQOL. Therefore, these results should be interpreted with caution and re-evaluated in future studies; we plan to include more study groups in a multicentre study. Replicating this study with more data and more robust study designs is warranted to confirm these results.

## Conclusions

The general health-related quality of life in the Polish population of patients after myocardial infarction is moderate and depends on several sociodemographic and clinical factors as well as on functioning in chronic disease**.** In relation to medical practice, the measurement of quality of life among patients after MI should be performed by all members of the treatment team due to its clinical implications. In clinical practice, the assessment of HRQoL should be considered a central indicator of the need for medical services, the effectiveness of therapeutic intervention and the effects of cardiac rehabilitation on patients after MI. This ability is diminished by some socioeconomic, psychological, cognitive and physical factors, and the incorporation of HRQoL measurement into clinical practice may make it easier to identify them. It is therefore reasonable to perform quality of life assessment as a holistic approach to post-MI care in healthcare policies.

## Data Availability

The datasets used and/or analysed during the current study available from the corresponding author on reasonable request.

## References

[1] The World Health Organization Quality of Life assessment (WHOQOL): Position paper from the World Health Organization. *Soc. Sci. Med*. **41**(10),1403–9 (1995). 10.1016/0277-9536(95)00112-k 8560308

[2] Sertoz OO (2013). The impact of physical and psychological comorbid conditions on the quality of life of patients with acute myocardial infarction: A multi-center, cross-sectional observational study from Turkey. Int. J. Psychiatry Med..

[3] Bahall M, Legall G, Khan K (2020). Quality of life among patients with cardiac disease: The impact of comorbid depression. Health Qual. Life Outcomes.

[4] Mills GB (2021). Is the contemporary care of the older persons with acute coronary syndrome evidence-based?. Eur. Heart J. Open.

[5] Disease burden and mortality estimates. *World Health Organization* https://www.who.int/news-room/fact-sheets/detail/the-top-10-causes-of-death (2020).

[6] Visseren, F.L.J. et al. ESC National Cardiac Societies; ESC Scientific Document Group. 2021 ESC Guidelines on cardiovascular disease prevention in clinical practice. *Eur Heart J.* **42** (34),3227–3337 (2021). 10.1093/eurheartj/ehab484. Erratum in: *Eur Heart J.* **7** 43(42),4468 (2022). 10.1093/eurheartj/ehab484 34458905

[7] Stepinska J (2020). Diagnosis and risk stratification of chest pain patients in the emergency department: Focus on acute coronary syndromes. A position paper of the Acute Cardiovascular Care Association. Eur. Heart J. Acute Cardiovasc. Care.

[8] Saeed T, Niazi GS, Almas S (2011). Type-D personality: A predictor of quality of life and coronary heart disease. East Mediterr. Health J..

[9] Wang W, Chow A, Thompson DR, Koh K, Kowitlawakul Y, He HG (2016). Predictors of health-related quality of life among patients with myocardial infarction. West. J. Nurs. Res..

[10] Westin L, Nilstun T, Carlsson R, Erhardt L (2005). Patients with ischemic heart disease: Quality of life predicts long-term mortality. Scand. Cardiovasc. J..

[11] Mori M, Djulbegovic M, Hajduk AM, Holland ML, Krumholz HM, Chaudhry SI (2021). Changes in functional status and health-related quality of life in older adults after surgical, interventional, or medical management of acute myocardial infarction. Semin. Thorac. Cardiovasc. Surg..

[12] Tegn N (2018). Health-related quality of life in older patients with acute coronary syndrome randomised to an invasive or conservative strategy. The After Eighty randomised controlled trial. Age Ageing.

[13] Bielawa Ł (2017). Evaluation of the quality of life of men undergoing stationary cardiac rehabilitation. J. Educ. Health Sport.

[14] Doll JA (2020). Quality of life after myocardial infarction: More PROgress needed. Heart.

[15] Decourcelle V, Maréchaux S, Pinçon C, Barrailler S, Le Jemtel TH, Ennezat PV (2013). Impact of functional decline on outcome in elderly patients with acute coronary syndromes. Am. J. Crit. Care.

[16] Halvorsen S (2022). 2022 ESC Guidelines on cardiovascular assessment and management of patients undergoing non-cardiac surgery. Eur. Heart J..

[17] Kang K, Gholizadeh L, Inglis SC, Han HR (2017). Correlates of health-related quality of life in patients with myocardial infarction: A literature review. Int. J. Nurs. Stud..

[18] von Elm E, Altman DG, Egger M, Pocock SJ, Gøtzsche PC, Vandenbroucke JP, STROBE Initiative (2014). The Strengthening the Reporting of Observational Studies in Epidemiology (STROBE) Statement: Guidelines for reporting observational studies. Int. J. Surg..

[19] Statistics Poland, Demographic Surveys Department. Demographic situation in Poland up to 2020. Death and mortality. https://stat.gov.pl/files/gfx/portalinformacyjny/pl/defaultaktualnosci/5468/46/1/1/sytuacja_demograficzna_polski_do_2020_r._zgony_i_umieralnosc._publikacja_w_formacie_pdf.pdf (2020).

[20] Skevington SM, Lotfy M, O’Connell KA (2024). The World Health Organization's WHOQOL-BREF quality of life assessment: Psychometric properties and results of the international field trial. A report from the WHOQOL group. Qual. Life Res..

[21] Jaracz K, Wołowicka L (2001). WHOQOL-BREF key. Quality of Life in Medical Sciences.

[22] Buszko K (2018). Validation of the functioning in chronic illness scale (FCIS). Med. Res. J..

[23] World Medical Association (2013). World Medical Association Declaration of Helsinki: Ethical principles for medical research involving human subjects. JAMA.

[24] Vijayananthan A, Nawawi O (2008). The importance of Good Clinical Practice guidelines and its role in clinical trials. Biomed. Imaging Interv. J..

[25] Lamesgin Endalew H (2022). Health-related quality of life among myocardial infarction survivors: structural equation modeling approach. J. Multidiscip. Healthc..

[26] Hawkes AL, Patrao TA, Ware R, Atherton JJ, Taylor CB, Oldenburg BF (2013). Predictors of physical and mental health-related quality of life outcomes among myocardial infarction patients. BMC Cardiovasc. Disord..

[27] Gąsecka A (2021). Health-related quality of life increases after first-time acute myocardial infarction: A population-based study. Zdr Varst..

[28] Rodríguez-Almagro J, García-Manzanares Á, Lucendo AJ, Hernández-Martínez A (2018). Health-related quality of life in diabetes mellitus and its social, demographic and clinical determinants: A nationwide cross-sectional survey. J. Clin. Nurs..

[29] Kolarić V, Svirčević V, Bijuk R, Zupančič V (2022). Chronic complications of diabetes and quality of life. Acta Clin. Croat.

[30] Mollon L, Bhattacharjee S (2017). Health related quality of life among myocardial infarction survivors in the United States: A propensity score matched analysis. Health Qual. Life Outcomes.

[31] Arnold SV, Masoudi FA, Rumsfeld JS, Li Y, Jones PG, Spertus JA (2014). Derivation and validation of a risk standardization model for benchmarking hospital performance for health-related quality of life outcomes after acute myocardial infarction. Circulation.

[32] Ogińska-Bulik N (2014). Type D personality and quality of life in subjects after myocardial infarction. Kardiol. Pol..

[33] Džubur A (2022). Relationship between depression and quality of life after myocardial infarction. Med. Glas. (Zenica)..

[34] Upadhyay V, Bhandari SS, Rai DP, Dutta S, García-Grau P, Vaddiparti K (2022). Improving depression and perceived social support enhances overall quality of life among myocardial infarction survivors: necessity for integrating mental health care into cardiac rehabilitation programs. Egypt. J. Neurol. Psychiatr. Neurosurg..

[35] French DP, Lewin RJ, Watson N, Thompson DR (2005). Do illness perceptions predict attendance at cardiac rehabilitation and quality of life following myocardial infarction?. J. Psychosom. Res..

[36] Steele A, Wade TD (2004). The contribution of optimism and quality of life to depression in an acute coronary syndrome population. Eur. J. Cardiovasc. Nurs..

